# Stress cardiac MR beyond ischemia - prevalence and characterization of previously unknown incidental findings with potential clinical implications

**DOI:** 10.1186/1532-429X-17-S1-P131

**Published:** 2015-02-03

**Authors:** Hugo Marques, António M Ferreira, Nuno Cardim, Pedro Gonçalves

**Affiliations:** FCM - UNL, Lisboa, Portugal; Radiology, UNICA - Hospital da LUZ, Lisbon, Portugal; UNICA - Hospital da Luz, Lisbon, Portugal

## Background

Adenosine stress cardiac MRI is a safe diagnostic test that enables evaluation beyond ischemia and viability. This quality is seldomly valued when cardiac MR is compared with other ischemic imaging tests (SPECT, PET and ECHO).

To assess the prevalence of significant non-ischemic incidental findings in patients undergoing adenosine stress cardiac magnetic resonance.

## Methods

We assessed 238 consecutive patients (154 male, mean age 64+/- 11 years) undergoing adenosine stress cardiac MR in a single center between January 2012 and July 2014.

Significant incidental results were defined as any previously unknown non ischemic finding with potential clinical and/or therapeutic implications (all patients had an previous cardiac ultrasound done within the previous 5 months).

The exams were performed on a 1.5T MR equipment and according to the SCMR guidelines for a adenosine stress cardiac MR.

Interpretation was done by agreement between a Radiologist and a Cardiologist with level III equivalent certification.

## Results

New significant non ischemic findings were detected in 34 patients (14.3%).

These finding were consistent with:

Hypertrophic cardiomyopathy (n=9); dilated cardiomyopathy with a non specific delayed enhancement (n=5); apical thrombus (n=2); lung nodules (n=3); thoracic aorta dilatation/aneurysm (n=3); chronic pulmonary thromboembolism (n=1); atrial septal defect (n=1).

There were also 10 cases of non ischemic delayed enhancement, 8 of them consistent with myocarditis sequelae.

Besides, we detected 7 previously unknown subendocardial myocardial infarcts.

All exams were concluded without any major complication (only one mild angioedema) and were judged with diagnostic quality.

## Conclusions

A significant proportion (14%, in our series) of patients undergoing stress cardiac MR has relevant previously unknown, non-ischemic findings, mostly related to cardiomyopathies.

The capacity of CMR to make these diagnoses in the same exam may be relevant when choosing an ischemic imaging test.

## Funding

N/A.

